# A commentary on ‘Incidence, risk factors, and outcomes of venous thromboembolism in patients undergoing surgery for retroperitoneal tumors: a propensity-matched retrospective cohort study’

**DOI:** 10.1097/JS9.0000000000001243

**Published:** 2024-03-04

**Authors:** Jiawen Liu, Zheng Jiang, Yefeng Yin

**Affiliations:** a Department of Gastrointestinal, The Ansteel Group Hospital, Anshan, Liaoning; b Department of Colorectal, Cancer Hospital Chinese Academy of Medical Sciences, Beijing, People’s Republic of China

*Dear Editor*,

Venous thromboembolism (VTE) consists of the interrelated diseases of pulmonary embolism (PE) and deep-vein thrombosis (DVT), which often occur after triggering factors, such as immobilization, trauma, surgery, cancer, or hospitalization^1^. Guo *et al*.^2^ conducted a propensity-matched retrospective cohort study on patients with retroperitoneal tumor surgery. In conclusion, VTE is strongly correlated with age, recurrence, and vascular resection, all of which are linked to a lower overall survival rate.

One thousand two hundred twenty-three patients were included in this retrospective cohort analysis, which examined the features, perioperative outcomes, and overall survival (OS) of patients (both VTE and non-VTE) who had retroperitoneal tumor surgery performed between 2015 and 2020. Perioperative and oncologic outcomes were compared using propensity-matching and Cox analyses. According to the study’s findings, 2.1% of the 1223 patients who had retroperitoneal tumor surgery experienced VTE. The results of multivariate analysis showed that age (OR=1.140, 95% CI: 1.053–1.239, *P*=0.004), recurrence (OR=1.851, 95% CI: 1.241–2.761, *P*=0.003), and vascular resection (OR=2.036, 95% CI: 1.054–3.934; *P*=0.034) were the factors related with VTE less than or equivalent to 90 days following surgery.

The data provide insight into short-term outcomes, which were compared between the VTE and non-VTE groups, with odds ratios (ORs) calculated using logistic regression to account for paired data. It shows that, with lower OS in VTE, the mean length of hospital stay and ICU stay were shorter in non-VTE patients than in VTE patients. However, there were numerous limits. First, biases like selection bias and unmeasured confounding might affect regularly gathered administrative health data^3^. The selection bias resulted from the fact that all of the data came from West China Hospital, Sichuan University, which was a top hospital. Second, because this is a retrospective study, there may be some bias, such as observer bias, reporting bias, and measuring bias. Third, the study solely differentiates VTE based on distance to the body, which may lead to bias. PE, DVT, and its subtypes – lower extremity, upper extremity, intra-abdominal, and intra-throacic – have distinct clinical implications^4^. To accurately interpret the data, each component of VTE being examined in the cohort must be defined clearly and consistently. The risk for PE and DVT, two different outcome metrics, should be quantified in a generalizable VTE risk assessment model. Finally, because the risk of VTE varies over time, it’s better to compare the risk of VTE in different follow-up times. Published publications have indicated their VTE rates vary from 1 day to as long as 180 days, and the Caprini RAM was established to predict VTE risk at 30 days. A more comprehensive and multicenter study is needed to corroborate this finding. Furthermore, further prospective research, clinical trials, and a study of indications and risk factors in diverse patient subgroups are required to determine the best course of action.

This study looks into the risk variables associated with individuals receiving retroperitoneal tumor surgery as well as the incidence of postoperative VTE in these patients. This issue has far-reaching implications for clinical practice. This large-scale retrospective cohort analysis provides preliminary evidence that age, recurrence, and vascular resection are all associated with VTE, which is associated with a poor prognosis, laying the groundwork for bigger, multicenter trials. The relative risks of short-term outcomes and long-term survival between patients with and without VTE are also discussed. This forward-thinking approach provides valuable guidance and inspiration for the continued development of this area of research. Higher-quality, prospective data is needed to establish a nomogram for predicting VTE to provide a reference for clinicians to identify people at high risk of VTE^5^.

## Ethical approval

This manuscript is a comment. Don’t need ethical approval.

## Consent

This manuscript is a comment. Don’t need patient consent.

## Author contribution

J.L.: study concept or design, data collection, data analysis or interpretation, and writing and revising the paper; Z.J.: study concept or design, data collection, data analysis or interpretation, and writing the paper; Y.Y.: data collection.

## Conflicts of interest disclosure

The authors declare no conflict of interest.

## Research registration unique identifying number (UIN)

This manuscript is a comment. Don’t need UIN.

## Guarantor

Jiawen Liu.

## Data availability statement

This manuscript is a comment. Don’t need a Data availability statement. However, all the data from the current study are publicly available.

## Provenance and peer review

This manuscript is a comment without being invited.
